# Astaxanthin Reduces Demyelination and Oligodendrocytes Death in A Rat Model of Multiple Sclerosis

**DOI:** 10.22074/cellj.2021.6999

**Published:** 2020-04-22

**Authors:** Alireza Lotfi, Mitra Soleimani, Nazem Ghasemi

**Affiliations:** Department of Anatomical Sciences, School of Medicine, Isfahan University of Medical Sciences, Isfahan, Iran

**Keywords:** Astaxanthin, Cuprizone, Multiple Sclerosis, Oligodendrocyte

## Abstract

**Objective:**

Astaxanthin (AST) is a carotenoid with anti-oxidative, anti-inflammatory, and anti-apoptotic properties. It has also been reported that AST exerts protective effects against neurodegenerative diseases and reduces oxidative stress-induced the central nervous system (CNS) injury. In this study, we aimed to evaluate the protective potential of AST in inhibiting demyelination and oligodendrocyte death in a rat model of multiple sclerosis (MS).

**Materials and Methods:**

In this experimental study, forty Wistar rats were randomly assigned to four experimental groups: control group (with normal feeding), cuprizone (CPZ group) that daily received 0.6% CPZ for 4 weeks, sham group that daily received 0.6% CPZ plus dimethyl sulfoxid (DMSO) for 4 weeks, and AST group that daily received 0.6% CPZ and after 12 hours were treated with AST (3 mg/kg), for 4 weeks. Muscle strength was evaluated by the behavioral basket test at the end of every week for 4 weeks. Luxol Fast Blue (LFB) staining was utilized for the identification of myelination and demyelination. Myelin density was evaluated by the ImageJ software. The expression of A2B5 (oligodendrocyte precursor protein) and myelin oligodendrocyte protein (MOG) were assessed by immunohistochemistry (IHC) and the expression of myelin basic protein (*MBP*), MOG, and platelet-derived growth factor-alpha (*PDGFR-α*) genes was examined by the real-time polymerase chain reaction (RT-PCR) technique.

**Results:**

The administration of AST reduced the oligodendrocyte damage and myelin sheath disruption in a rat model of MS. The basket behavioral test showed the improvement of muscle strength in the AST group compared with CPZ and sham groups. Besides, the results of real-time PCR and IHC indicated the beneficial effects of AST in declining demyelination and oligodendrocyte death in a rat model of MS.

**Conclusion:**

AST reduces damages to the myelin sheath and oligodendrocyte death in a rat model of MS.

## Introduction

Neurodegeneration is a feature of several debilitating and progressive disorders characterized by chronic loss of neurons in the nervous system. Demyelination is one of the most important causes of neurological disability (1, 2). Multiple sclerosis (MS) is a chronic inflammatory disease characterized primarily by demyelination and progressive neurodegeneration in the central nervous system (CNS). Oligodendrocyte death due to focal immune cell infiltration is the primary cause of demyelination and has an important role in the pathogenesis of MS (3). Hence, the prevention of oligodendrocyte death by the use of natural compounds, such as curcumin (4) and AST may decrease the adverse complications developed in demyelinating disorders.

AST (3, 3’-dihydroxy-ß, ß’-carotene-4, 4’-dione) is a natural red fat-soluble xanthophyll carotenoid produced by marine microorganisms, gaining considerable attention by researchers in recent years. Studies examined the protective properties of AST indicate that its protective roles are due to three main effects: anti-oxidative, antiinflammation, and anti-apoptotic effects. It has been reported that AST has anti-oxidative properties ten times more than some carotenoids, such as lutein, canthaxanthin, and zeaxanthin, as well as a hundred times more than vitamin E (5).

In addition, other experiments demonstrated that AST has a protective impact against primary brain damages, as well as the destruction of neurons and blood-brain barrier (BBB) disruption, cerebral edema, and impaired nerve function through suppressing brain inflammation (6).

Additional works also demonstrated other effects of AST, including anti-lipid peroxidation and anticancer activity (7, 8).

These protective properties mostly pertain to the unique chemical structure of AST, the presence of hydroxyl and keto moieties on each ion ring. This unique nature confers the ability to AST to be esterified, posing a higher antioxidant activity and a more polar nature than other carotenoids (9). Neuroprotective properties of AST have been evaluated in animal models, and its protective roles have been addressed by several research groups (5, 10-13). Some parts of these beneficial roles are attributed to the ability of AST in crossing the BBB (10).

Utilizing animal models of neurodegenerative diseases has been enhancing the knowledge of molecular pathogenesis which is responsible for the onset and development of MS and other potentially disabling neurodegenerative disorders (14). Among different protocols that have been introduced for producing an animal model of MS, CPZ is the well-stablished one to study demyelination and remyelination in rodent models of MS (15).

In this study, we designed an experiment to assess the impact of AST in the amelioration or prevention of demyelination in a rat model of MS.

## Materials and Methods

### Ethics statement

This experimental study was performed at the Isfahan University of Medical Sciences (Isfahan, Iran). All experimental procedures were conducted in compliance with the guidelines of the Iranian Committee of Animal care and approved by the Ethics Committee of Isfahan University of Medical Sciences (Ethics#IR.MUI.MED.REC.1398.037).

### Animals

Forty male Wistar rats, weighing 150-200 g, were purchased from the Royan Institute, Isfahan, Iran. Rats were housed for seven days, before the start of the experiment, in constant environmental conditions, at a temperature of 22 ± 2˚C with free access to food and water and a 12:12 light/dark cycle.

### Behavioral test

The Basket behavioral test evaluates motor coordination and sensorimotor deficits in rodent models of CNS disorders (4). For this purpose, rats were placed in the center of a rectangular basket (60 cm in length and 50 in width), and then the basket was overturned and the delay in dropping rats from the ceiling of the basket was determined over 180 seconds, and eventually the mean of these times (seconds) were recorded. The experiment was conducted triplicate.

### Production of a rat model of multiple sclerosis

Animals were randomly divided into four groups. The number of rats per group was determined by ten rats, by means of the power analysis calculations. To produce a rat model of MS, the CPZ suspension was prepared by the use of CPZ powder (Sigma-Aldrich, USA) and 1% methylcellulose (Sigma-Aldrich, USA). To prepare AST (Sigma-Aldrich, USA), AST powder were dissolved in dimethyl sulfoxid (DMSO, Gibco, USA) to reach a homogenous solution. Rats were fed as planned per group. The control group was fed with normal chow for four weeks. The CPZ group was treated with 2 ml CPZ (0.6%), by gavage, daily for 4 weeks (16). The sham group was first treated with 2 ml CPZ (0.6%) and after 12 hours treated with 2 ml DMSO, by gavage, daily for 4 weeks (this was done to reject any possibility of repairmen effects of DMSO as the solvent of AST). The AST group first received CPZ 0.6% and after 12 hours received 2 ml AST (3 mg/kg/day) (17), daily for 4 weeks. Animals in all groups were weighed 3 times per week during chemical administrations until they were sacrificed. Tissue samples from all groups were processed at the same time within the same experiment.

### Craniotomy and dissection of the corpus callosum

Animals were sacrificed by performing deep anesthesia using ketamine/xylazine (100/10 mg/kg, Sigma-Aldrich, Germany) and decapitated. The brains were removed by craniotomy of the vertex, weighed precisely and then immersed in the fixative solution of formaldehyde and formalin (10%, Sigma-Aldrich, USA) for more than 24 hours. After washing with a 0.1 M phosphate buffer (Sigma-Aldrich, USA), the brains were sectioned at the coronal plane and using its natural cleavage plane, the corpus callosum identified and dissected, removing samples of the mid-body. After tissue processing, the blocks were cut in sections of 5- and 6-ىm of thickness, and these sections were used for Luxol Fast Blue (LFB) staining and immunohistochemistry technique.

### Immunohistochemistry

After tissue processing by use of ethanol, xylene and paraffin (Asia Pajohesh, Iran), serial sections (5- μm thick) of the brain samples were prepared using a microtome. After de-paraffinization and rehydration, the slices were pre-treated using the method of heatinduced epitope retrieval with sodium citrate buffer (pH=6) and 1 mM EDTA buffer (pH=8) (Gibco, USA) for 20 minutes, washed-out three times with PBS (Gibco, USA). Then, the slices were incubated with special antibodies against A2B5, a marker of oligodendrocyte precursor cells (Abcam, USA) and MOG, a myelin oligodendrocyte glycoprotein (Abcam, USA), and then washed-out with PBS. Then, goat antimouse -FITC (2 μg/ml, Abcam, USA) and rabbit antigoat- FITC (Sigma-Aldrich, USA) were used as the secondary antibody at room temperature for 1 hour. Finally, nuclear counterstaining was conducted using 4, 6 Diamino-2-phenylindole, dilactate (DAPI) and in order to quantitative analysis, the total numbers of positive cells were counted in a minimum total of 200 cells per slide in six sections per sample using .uorescence microscope (Olympus bx51, Japan). Meanwhile, all IHC analyses were repeated at least three times.

### Luxol Fast Blue staining

After deep anesthesia, perfusion fixation was done by use of cold PBS and 4% paraformaldehyde. In the following, corpus callosum dissection was performed, and in order to post-.xation, formalin solution (10%) was used at 4°C overnight. Subsequently, paraffin slices (6 µm) were prepared. Then sections were dewaxed and cleared in 100% and 95% ethanol and then were stained in a solution of LFB (0.1% w/v, Sigma- Aldrich, USA), 10% acetic acid and ethanol (95%, Asia Pajohesh, Iran) overnight at 56°C. Sections then were differentiated by rinsing in 95 % ethanol, 0.05% lithium chloride solution (Asia Pajohesh, Iran) followed by 70 % ethanol. Differentiation continued in distilled water until unmyelinated tissue appeared white (18).

### Gene expression analysis

In order to evaluate gene expression, real-time polymerase chain reaction (RT-PCR) analysis of *MOG*, *MBP*, and *PDGFR-α* was performed according to the SYBR Green master mix protocol. According to our previous study, Total RNA was extracted from the brain corpus callosum by using RNeasy micro kit (Qiagen, GmBH, Germany) and then, 2 μg of RNA was used for cDNA synthesis using RevertAid First Strand cDNA Synthesis Kit (Fermentas, Germany) (19). Finally, quantitative real-time PCR was done using Thermal Cycler Rotor-Gene in a total volume of 20 μl containing Power SYBR Green master mix (2x), forward and reverse Primers (0.5 μM), cDNA (30 ng/μl) and H2O with the following cycling conditions: 1 cycle of denaturation (95˚C for 5 minutes) which followed by a 40 cycle amplification (at 95˚C for 15 seconds) and the extension cycle (at 60˚C for 40 seconds). The primer sequences used for RT-PCR analysis in this study are listed in Table 1.

**Table 1:** Primer sequences


Gene primers	Primer sequence (5ˊ-3ˊ)

*PDGFR-α*	F: tccagtcactgtgctgcttc
	R: gcaagggaaaagggagtctt
*MOG*	F: gaggttctcggatgaaggag
	R: cagggttgatccagtagaagg
*MBP*	F: tcacagaagagaccctcacag
	R: ggtgtacgaggtgtcacaatg
*GAPDH*	F: tgcaccaccaactgcttagc
	R: ggcatggactgtggtcatgag


### Data analysis

Immunohistochemistry, behavior test, RT-PCR, and LFB results were analyzed using the SPSS software version 25.0 (SPSS Inc., Chicago, IL, USA). One-way ANOVA was conducted, followed by Tukey’s post hoc test. In addition, all data were shown as mean ± standard error of the mean (mean ± SEM).

## Results

### Basket behavior test

**Fig.1:**
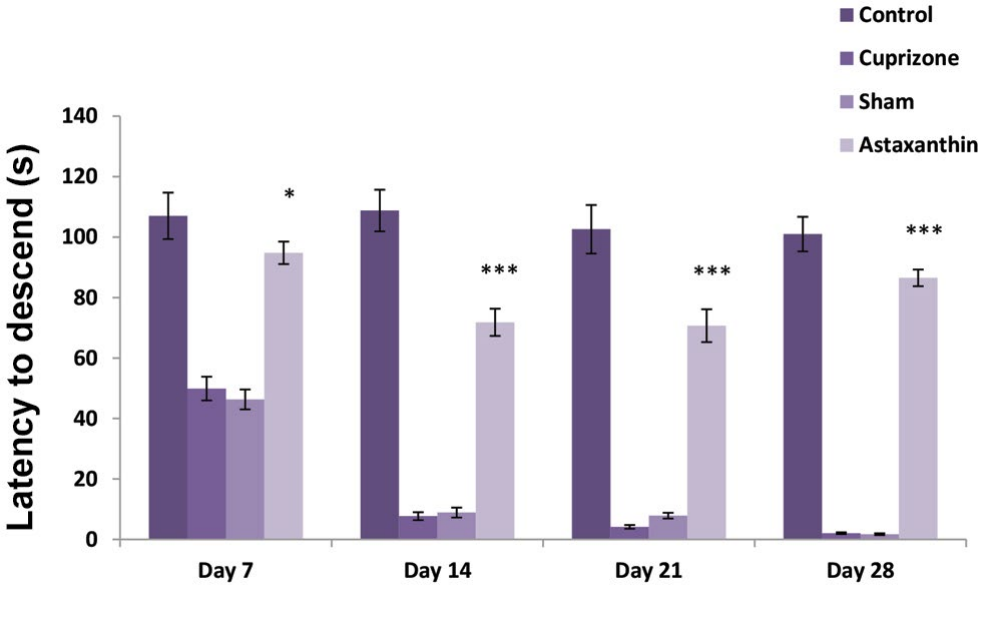
The comparison of latency to fall in different groups. In the Cuprizone and sham groups, the average latency to fall was decreased significantly from the first week in comparison with the Astaxanthin group (P<0.05). *; P<0.05 and ***; P<0.001.

The basket behavioral test was performed at the end of every week for a total of 4 weeks. During this study, the comparison of behavioral results demonstrated that the use of AST increases the average latency to fall compared to sham and CPZ groups. Unlike the AST group, in CPZ and sham groups, the average latency to fall was decreased significantly from the first week (P≤0.05, Fig.1).

### Histological observation of cuprizone-induced demyelination in rats

LFB staining of coronal sections of corpus callosum observed under ×40 magnification. Myelinated axons were stained blue. Achievement of MS induction in rat models was confirmed by prominent demyelination in CPZ group and vehicle group. While the control group represented blue colored bands indicating the presence of myelinated axons (Fig.1). In the AST group, majority of axons stained blue, while few axons were stained pink, confirming the inhibitory effects of AST in demyelination.

**Fig.2:**
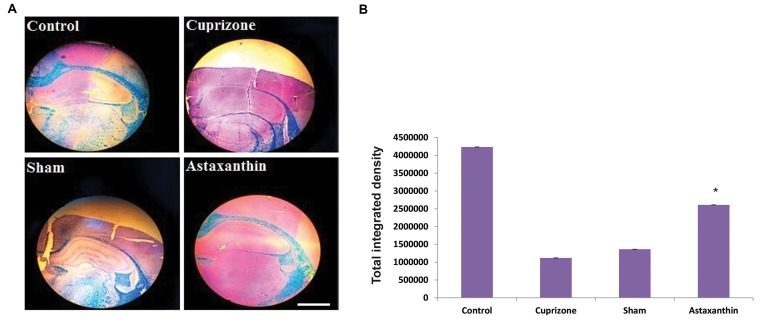
Illustration of LFB in coronal sections of the brain of Wistar rats to assess myelin status, after treatment with CPZ. **A.** The blue colured region indicates the presence of myelin. In the control group, a distinct delineated pattern of areas without (pink) and with stained myelin (blue) is observable. In CPZ and sham groups, there is a significant reduction in the myelin contents in the corpus callusom, indicated by an asterisk. In the AST group, induced with CPZ and treated with AST, a sharply delineated pattern is identifiable.** B**. The total integrated density of myelin calculated by ImageJ software (scale bar: 200 μm). LFB; Luxol Fast Blue, CPZ; Cuprizone, AST; Astaxanthin, and *; P<0.05.

Moreover, calculating the myelin density by the ImageJ software, revealed that the myelin density of corpus callosum in tissue sections of the AST group was significantly higher than that of CPZ and sham groups (Fig.2).

### Immunohistochemistry

IHC results indicated that the mean percentage of positive cells for MOG and A2B5 proteins were significantly higher in the AST group compared with the CPZ group, 56 ± 2.7 and 59.7 ± 5.2, respectively (Figes.3, 4).

**Fig.3:**
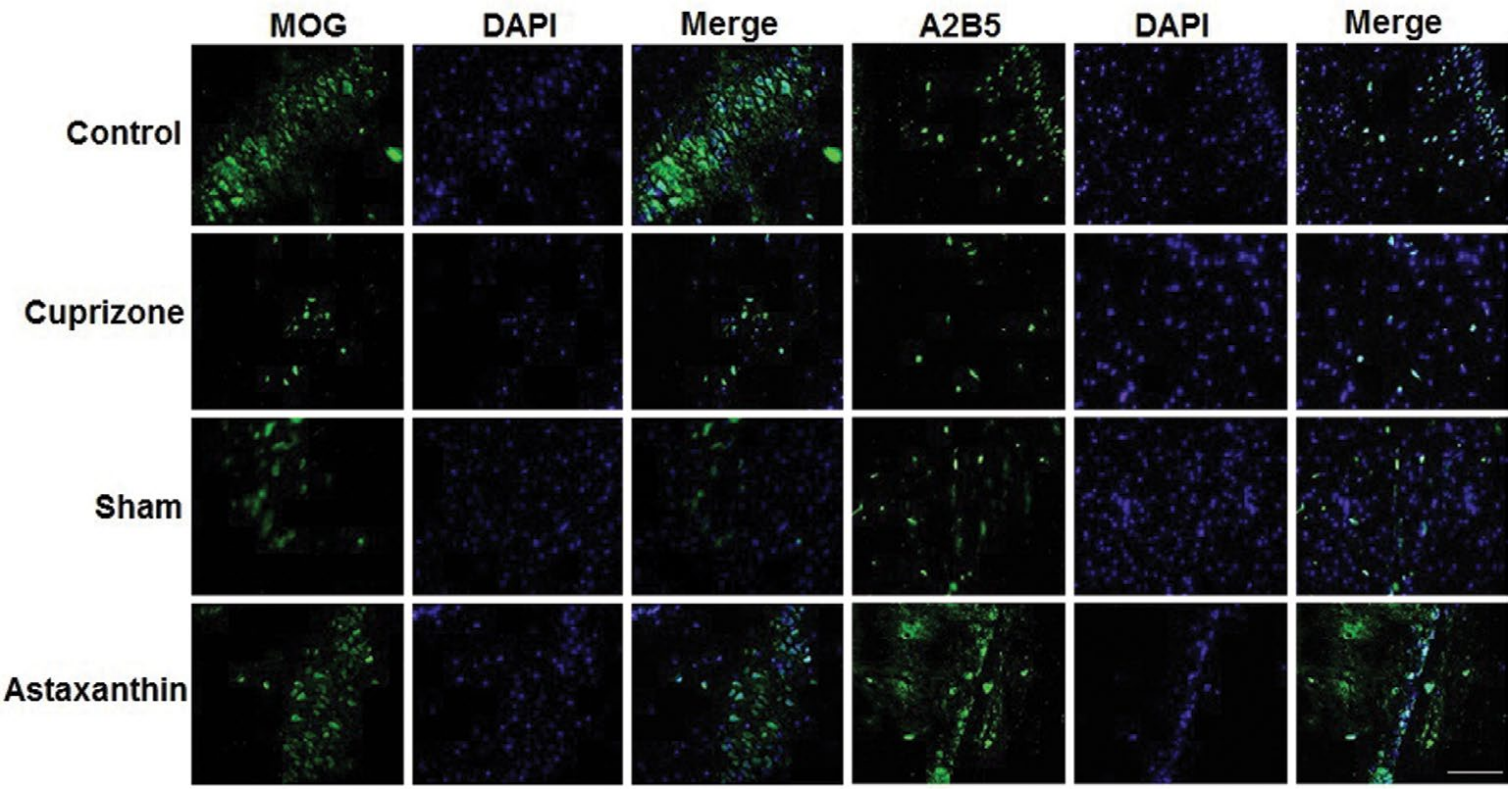
MOG and A2B5 stained of the corpus callosum in control, Cuprizone, sham, and Astaxanthin groups. The statistical analysis of MOG and A2B5 staining according to the relative optical density. The presence of MOG-positive cells (green) and A2B5 positive cells (green) were signiﬁcantly higher in Astaxanthin group as compared to Cuprizone and sham groups. Astaxanthin alleviated demyelination in Cuprizone-induced rat corpus callosum (scale bar: 200 μm).

**Fig.4:**
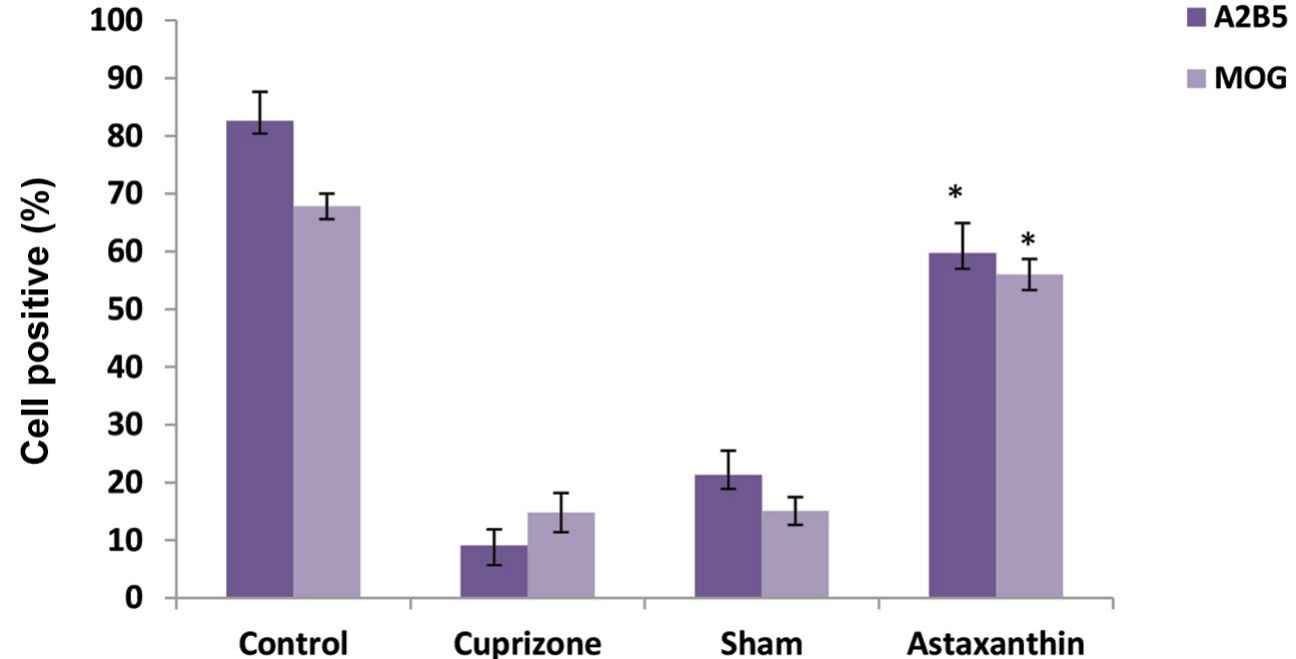
The comparison of A2B5 and MOG expression in all experimental groups. In the Astaxanthin group, the mean percentage of cells which express A2B5 and MBP markers was statistically significant in comparison with Cuprizone and sham groups (P<0.05). *; P<0.05.

### Gene expression analysis

**Fig.5:**
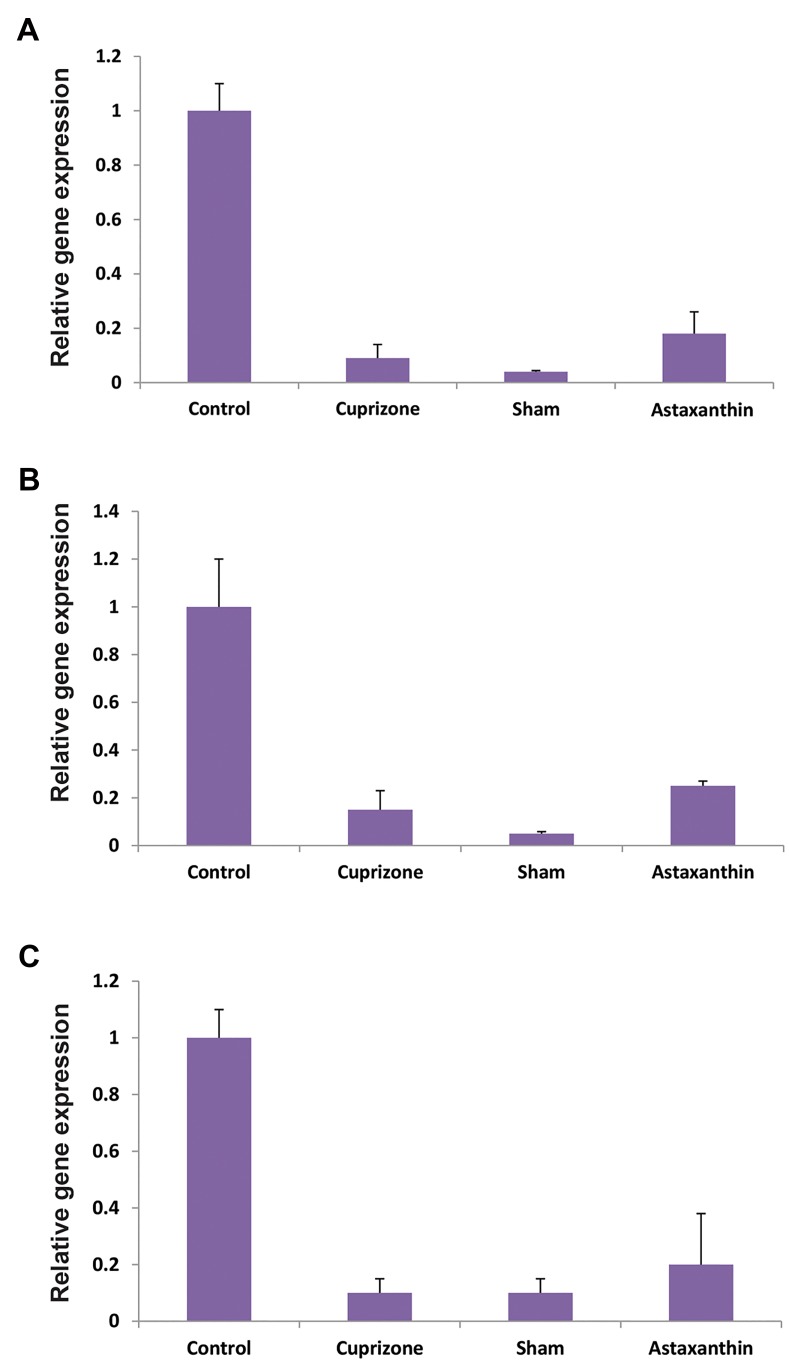
Comparative analysis of *MBP, MOG* and *PDGFR-α* markers in different groups. The relative expression levels of **A.** *MBP*, **B.** *MOG*, and **C.** *PDGFR-α* genes that were evaluated by the real-time polymerase chain reaction. The expression of all three genes increased significantly in the Astaxanthin group compared to Cuprizone and sham groups.

Real-time PCR results of gene expression analysis in four experimental groups indicated that mRNA expression of the *MOG*, *MBP*, and *PDGFR-α* genes were higher in AST group compared to CPZ group and the sham group that represented similar expression pattern for these genes (Fig.5).

## Discussion

We concluded that consumption of AST modulated demyelination and oligodendrocyte death as well as muscle weakness in CPZ-induced rat model of MS. MS is a chronic inflammatory CNS disease leading to primary demyelination is featured with focal plaques (20). Some natural products already have been suggested to contribute to the alleviation of demyelination in demyelinating diseases (21). However, the mechanism of their effects rarely confirmed scientifically. AST is a natural product of aquatic microorganisms that recently has gained strong attention from medical and life science research (5). It is a lipophilic terpene and a metabolite of zeaxanthin. The presence of the hydroxyl and keto moieties on each ionone ring explains some of its unique features, namely, the ability to be esterified, higher antioxidant activity and a more polar nature than other carotenoids and so, AST is considered as a suitable multi-target pharmacological agent (9, 22). Due to its unique chemical structure, AST can cross the BBB (23). This property has conferred AST particular attention in research implementing in the realm of neurodegenerative disorders. Moreover, since the late onset of extensive neuronal death in neurodegenerative diseases is related to oxidative damage, and due to proven anti-oxidative effects of AST (11), CNS is considered one of its key target organs and has been as particular interest as co-treatments in neurodegenerative diseases. Therefore, in the present study, in order to investigate the neuroprotective effects of AST, the composition of the CPZ was used for induce a toxic model of MS and finally, the laboratory tests were performed on brain tissue.

CPZ (oxalic acid bis-cyclohexylidene hydrazide), is a copper chelator that induces highly reproducible demyelination in the CNS and extensively used to create a model of demyelinating disease (24). By the third week of CPZ treatment, consistent demyelination can be observed in the corpus callosum, the largest white matter tract in the rodent brain (25). So, in order to induce MS in rats, we treated male Wistar rats with 0.6% CPZ, daily for four weeks.

LFB stained sections displayed corpus callosum with defined borders of the corpus callosum, and there was a sharply delineated differentiation between white (blue) and gray matter (pink) in the control group. However, in CPZ-induced groups, CPZ and Sham, the myelin density, evaluated with the ImageJ software was shrunk, and no defined borders were observable in the stained samples. Four weeks after treatment with AST, the sections of LFB staining represented a corpus callosum that was significantly well- delineated and with cohesive texture compared to the shrunk texture were seen in CPZ group and Sham group. In justifying this result, it can be said that AST due to cross the BBB is able to carry out its neuroprotective effects at high levels and thus prevent myelin destruction. Another relevant finding of the current study was that AST is able to induce a significant delay in climbing down the basket wire wall in comparison to CPZ and sham groups. This result is consistent with a recent study which has also shown that AST can improve sensorymotor function through modulating specific cell signaling pathways in a rat model of spinal cord injury (26). For justifying this outcome, it can be said that AST is capable of improving the neural dysfunction, caused by CPZ by preventing demyelination and increasing the transmission of neural impulses. Finally, we evaluated the influences of CPZ and AST on oligodendrocytes. Progenitor cells of oligodendrocytes are widely scattered in CNS and are a constant reservoir for oligodendrocyte replacement and remodeling. To detect impairment effects of CPZ, and repairment influences of AST on rat model of MS, we stained corpus callosum section, in all our four groups, with two oligodendrocyte proteins, MOG and A2B5. MOG, myelin oligodendrocyte glycoprotein, expressed on the outer membrane of myelin sheath and oligodendrocytes, and exclusively found within the CNS. Giving the late commencement of MOG expression in myelination, it is believed that MOG plays a role in the maintenance of the myelin sheath and may serve as a potential marker in oligodendrocyte maturation. Therefore, MOG could potentially function as a cell surface marker of matured oligodendrocytes (27). During OPC differentiation, cells sequentially express A2B5, PDGFR-α, and MBP (28). Our stained sections of corpus callosum for MOG and A2B5 represented significant positive staining for both antibodies in the AST group than CPZ and sham groups. This may underpin the ameliorative effects of AST on oligodendrocyte death or even its oligodendrogenic effects. To confirm the obtained IHC results, we assessed the real-time PCR expression of *MOG*, *MBP*, and *PDGFR-α* genes in the corpus callosum in all groups. The results demonstrated that the expression of all three genes decreased significantly in CPZ and sham groups than the control group, indicating the destructive effects of CPZ in a rat model of MS.

Moreover, the expression of all three genes was higher in AST group compared to the CPZ and sham groups. This may represent that AST due to its antioxidant and neuroprotective effects, is capable of preventing the death of oligodendrocytes which induced by CPZ. Besides, since MOG and PDGFR-α have a sequential expression pattern in oligodendrocyte maturation, it could be suggested that AST may infuse oligodendrogenesis from progenitors to mature oligodendrocytes.

## Conclusion

AST may reduce demyelination and oligodendrocyte death in MS rat model. Either AST stimulates the proliferation of oligodendrocytes, or it prohibits death of pre-existing oligodendrocyte is an issue to be confirmed.
